# Sphingosine-1-phosphate in the lymphatic fluid determined by novel methods

**DOI:** 10.1016/j.heliyon.2016.e00219

**Published:** 2016-12-22

**Authors:** Masayuki Nagahashi, Akimitsu Yamada, Tomoyoshi Aoyagi, Jeremy Allegood, Toshifumi Wakai, Sarah Spiegel, Kazuaki Takabe

**Affiliations:** a Division of Digestive and General Surgery, Niigata University Graduate School of Medical and Dental Sciences, Niigata City, 951-8510, Japan; b Division of Surgical Oncology, Department of Biochemistry and Molecular Biology, and the Massey Cancer Center, Virginia Commonwealth University School of Medicine, Richmond, Virginia 23298-0011, USA; c Department of Biochemistry and Molecular Biology, and the Massey Cancer Center, Virginia Commonwealth University School of Medicine, Richmond, Virginia 23298-0011, USA; d Breast Surgery, Department of Surgical Oncology, Roswell Park Cancer Institute, Buffalo, NY 14263, USA; e Department of Surgery, University at Buffalo, The State University of New York, Jacobs School of Medicine and Biomedical Sciences, New York

**Keywords:** Surgery, Biochemistry, Cell biology, Immunology, Oncology, Cancer research

## Abstract

**Background:**

Sphingosine-1-phosphate (S1P) is a pleiotropic bioactive lipid mediator that regulates many physiological and pathological processes. It has been suggested that S1P gradient with high concentrations in the blood and lymphatic fluid and low concentrations in the peripheral tissue plays important roles in immune cell trafficking and potentially cancer progression. However, only a few reports have assessed S1P levels in the lymphatic fluid due to lack of an established easy-to-use method. Here, we report a simple technique for collection of lymphatic fluid to determine S1P.

**Materials and methods:**

Lymphatic fluid was collected directly with a catheter needle (classical method) or was absorbed onto filter paper after incision of cisterna chyli (new method) in murine models. Blood, lymphatic fluid and mesenteric lymph nodes were corrected from wild type and sphingosine kinase 2 (SphK2) knockout mice to determine S1P levels by mass spectrometry.

**Results:**

The volume of lymphatic fluid collected by the new method was at least three times greater than those collected by the classical method. S1P concentrations in lymphatic fluid are lower than in blood and higher than in lymph nodes. Interestingly, S1P levels in lymphatic fluid from SphK2 knockout mice were significantly higher than those in wild type, suggesting an important role of SphK2 and/or SphK1 to regulate S1P levels in lymphatic fluid.

**Conclusions:**

In agreement with the previous theory, our results confirm “S1P gradient” among blood, lymphatic fluid and peripheral lymphatic tissues. Convenient methods for collection and measurement of sphingolipids in lymphatic fluid are expected to provide new insights on functions of sphingolipids.

## 1 Introduction

Sphingosine-1-phosphate (S1P) is a pleiotropic bioactive lipid mediator that regulates many physiological and pathological processes including immune cell trafficking, inflammation, angio-/lymphangiogenesis, and cancer progression [1, 2, 3, 4, 5, 6]. We have recently reported that S1P is strongly associated with lymphatic network development [7, 8] and lymphatic metastasis in cancer patients [9, 10, 11]. Further, we have shown that S1P links inflammation and cancer in colitis-associated colon cancer [12, 13]. These many pathological processes are regulated by S1P that is secreted from cells to the extracellular spaces and exerts its functions by binding to five specific G protein–coupled receptors (S1PR1-5) in autocrine and paracrine manners, a process known as “inside-out” signaling [1, 14, 15].

S1P is generated by two sphingosine kinases (SphK1 and SphK2) inside cells, and secreted out of the cell to the extracellular spaces, such as interstitial fluid (IF) and lymphatic fluid [16]. Among the extracellular S1P, the S1P levels in the blood and lymphatic fluid are considered to be high, and those in tissue interstitial fluid are much lower [16]. Previous evidence suggested that the S1P gradient of high S1P concentrations in the circulation (blood and lymphatic fluid) plays an important role in immune cell trafficking [7, 17, 18, 19, 20] and potentially cancer progression. Lymphocytes egress from the secondary lymphatic organs to the blood or lymphatic circulation through S1P-mediated activation of S1PR1 on the cell surface [21, 22]. SphK activity in lymphatic endothelial cells is required for lymphocyte egress from secondary lymphatic organs, and lymphatic endothelial cells have been considered to be a major source of S1P in lymphatic fluid [23, 24].

Although S1P levels in blood have been published to be associated with lymphatic metastasis by our group and others [9, 16], only a few reports have assessed its levels due to lack of an established easy-to-use method. Considering the importance of S1P in lymphatic fluid for immune cell trafficking and cancer progression, establishing the easier method to measure it will help researchers to understand disease processes and move the research field forward. Here, we report a simple technique for collection of lymphatic fluid to determine sphingolipids including S1P by high sensitivity liquid chromatography-electrospray ionization tandem mass spectrometry (LC-ESI-MS/MS).

## 2 Material and methods

### 2.1 Reagents

Internal standards were purchased from Avanti Polar Lipids (Alabaster, AL) and added to samples in 20 μl ethanol:methanol:water (7:2:1) as a cocktail of 500 pmol each. The HPLC grade solvents were obtained from VWR (West Chester, PA).

### 2.2 Animals

All animal studies were conducted in the Animal Research Core Facility at VCU School of Medicine in accordance with institutional guidelines. We utilized SphK2 knockout mice as models since they are well characterized, and because we have previously found that SphK2 knockout mice demonstrate compensatory higher expression of SphK1 in the tissues [12]. These knockout mice were kindly given by Dr. Richard L. Proia of National Institute of Diabetes and Digestive and Kidney Diseases [25, 26]. Female C57BL/6 and SphK2 knockout mice and their corresponding wild type (WT) litter mates were used.

### 2.3 Collection of lymphatic fluid from the cisterna chyli

**Fig. 1:**
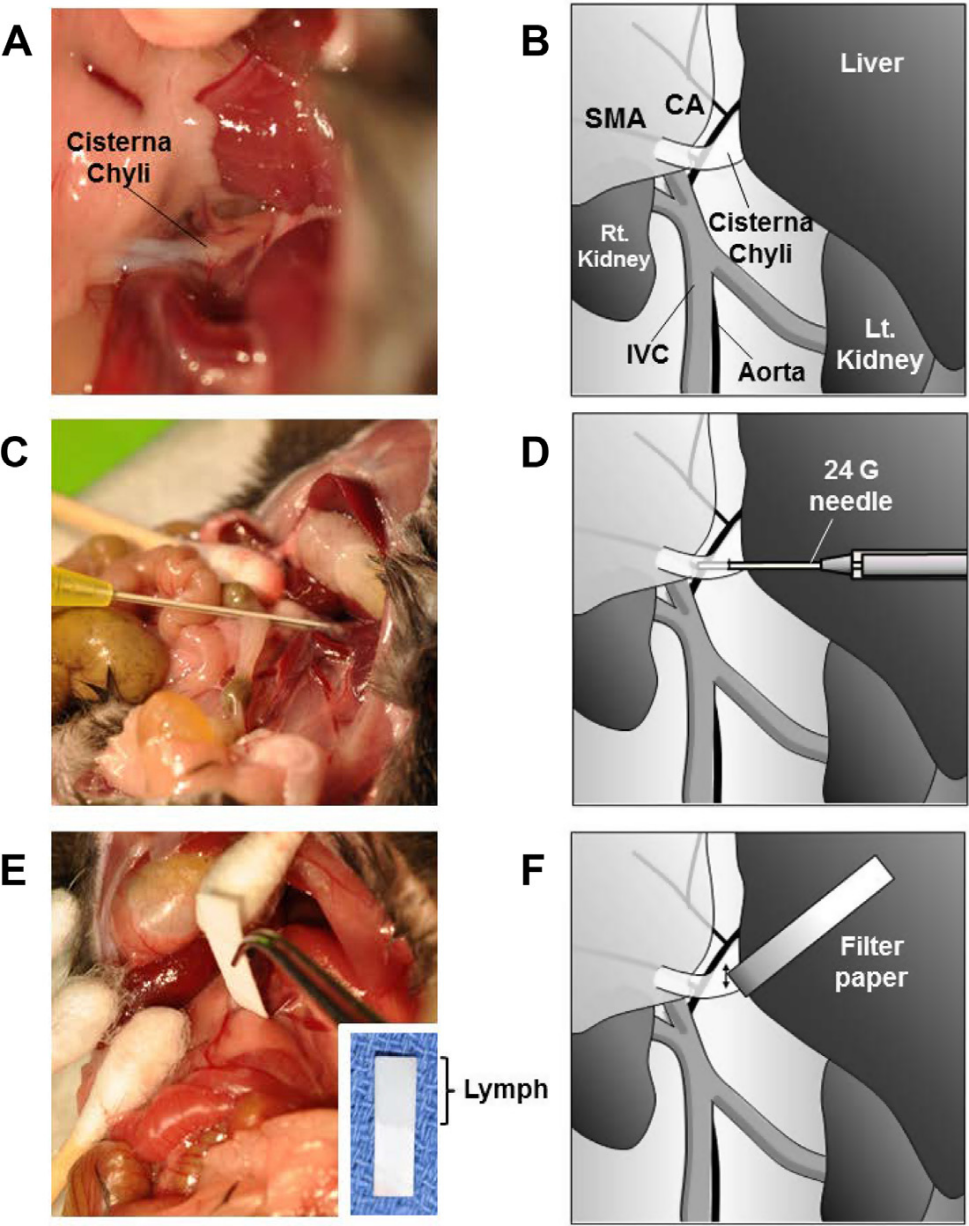
Collection of lymphatic fluid from a mouse. (A) Lymphatic fluid was observed as fluid with white lucent color in the intestinal trunk and the cisterna chyli. (B) Schematic illustration of lymph collection from a mouse. (C and D) Lymphatic fluid was collected directly with a 24 G catheter needle. (E and F) Lymphatic fluid was absorbed onto filter paper after incision of cisterna chyli by needle. CA, celiac artery; SMA, superior mesenteric artery; IVC, inferior vena cava.

To increase the lymphatic flow in the abdomen, 200 μl of mineral oil was administered by gavage to mice 2 h prior to euthanasia. At that time, the abdomen was opened under anesthesia. Lymphatic flow was confirmed by the white lucent color in the intestinal trunk and the cisterna chyli under stereomicroscopy (Fig. 1). The intestinal trunk is formed by the confluence of the efferent vessels of the cranial mesenteric and celiac nodes, and the single trunk enters into the cisterna chyli located along the abdominal vena cava and aorta on the cranial side of the renal veins [27]. The cisterna chyli was cut with the bevel of a fine needle, and the lymphatic fluid was absorbed with a piece of pre-weighed absorbance paper with a fine and rigid point. The absorbance paper was handled with extreme care in order to avoid contamination from any other intra-peritoneal fluid. This procedure was performed by experienced surgeons, surgical residents, or laboratory technicians. The volume of the collected fluid was measured by weight and sphingolipids were analyzed by LC-ESI-MS/MS.

### 2.4 Preparation of blood samples

Whole blood was collected from inferior vena cava (IVC), and 40 μl immediately transferred into 500 μl of methanol in a glass tube for measurement of sphingolipids. The remaining collected blood from the IVC was left undisturbed at room temperature, allowing the blood components to coagulate. Serum was then collected by centrifuging at 2,600 x g for 10 min in a refrigerated centrifuge.

### 2.5 Extraction of lipids

Fluid absorbed onto filter paper was placed in 13 × 100 mm borosilicate tubes with a Teflon-lined cap (VWR, West Chester, PA). Methanol (1 ml) and chloroform (0.5 ml) were added along with the internal standard cocktail. The contents were dispersed by sonication at room temperature for 30 s and incubated at 48 °C overnight. KOH (1 M) in methanol (75 μl) was added and, after brief sonication, samples were incubated in a shaking water bath for 2 h at 37 °C to hydrolyze glycerophospholipids. The extract was brought to neutral pH with 6 μl of glacial acetic acid, centrifuged using a table-top centrifuge, and the supernatant was removed and dried in a Speed Vac. The dried residue was reconstituted in 0.5 ml of the starting mobile phase solvent, briefly sonicated and then centrifuged for 5 min in a tabletop centrifuge before transfer of the clear supernatant to the autoinjector vials.

### 2.6 LC-ESI-MS/MS of sphingoid bases, sphingoid base 1-phosphates, and complex sphingolipids

For LC-ESI-MS/MS analyses, a Shimadzu LC-20 AD binary pump system coupled to a SIL-20AC autoinjector and DGU20A3 degasser coupled to an ABI 4000 quadrupole/linear ion trap (QTrap) (Applied Biosystems, Foster City, CA) operating in a triple quadrupole mode was used. Q1 and Q3 were set to pass molecularly distinctive precursor and product ions (or a scan across multiple m/z in Q1 or Q3), using N2 to collisionally induce dissociations in Q2 (which was offset from Q1 by 30–120 eV).

Sphingolipids were separated by reverse phase LC using a Supelco 2.1(i.d.)x50 mm Ascentis C18 column (Sigma, St. Louis, MO) and a binary solvent system at a flow rate of 0.5 ml/min. Prior to injections, the column was equilibrated for 0.5 min with a solvent mixture of 95% mobile phase A1 (CH_3_OH/H_2_O/HCOOH, 58/41/1, v/v/v, with 5 mM ammonium formate) and 5% mobile phase B1 (CH_3_OH/HCOOH, 99/1, v/v, with 5 mM ammonium formate), and after sample injection (typically 40 μl), the A1/B1 ratio was maintained at 95/5 for 2.25 min, followed by a linear gradient to 100% B1 over 1.5 min, which was held at 100% B1 for 5.5 min, followed by a 0.5 min gradient return to 95/5 A1/B1. The column was re-equilibrated with 95:5 A1/B1 for 0.5 min before the next run.

### 2.7 Statistical analysis

Results were analyzed for statistical significance with a two-tailed Student’s t-test, with *P* < 0.05 considered significant. Experiments were repeated at least three times. *In vivo* experiments were repeated three times and each experimental group consisted of at least four mice. Data presented is from one of three representative experiments.

## 3 Results

### 3.1 Improved method of lymphatic fluid collection from cisterna chyli

It has been suggested that S1P gradient with high concentrations in the blood and lymphatic fluid and low concentrations in the peripheral tissue plays important roles in immune cell trafficking and potentially cancer progression. However, only a few reports have assessed S1P levels in the lymphatic fluid due to lack of an established easy-to-use method. The classical method for collection of lymphatic fluid require lengthy duration of cannulations. Therefore, we sought to develop a simple method that does not require special equipment and settings. To this end, we compared collection of lymphatic fluid from the cisterna chyli by direct aspiration using a 24-gauge needle (classical method) with a filter paper absorption method after opening the cisterna chyli by incision with the bevel of a needle after adequate exposure of the lymphatic vessel (new method) (Fig. 1). The paper that absorbed lymphatic fluid was colorless indicating that there was little or no contamination with blood (Fig. 1E).

**Fig. 2:**
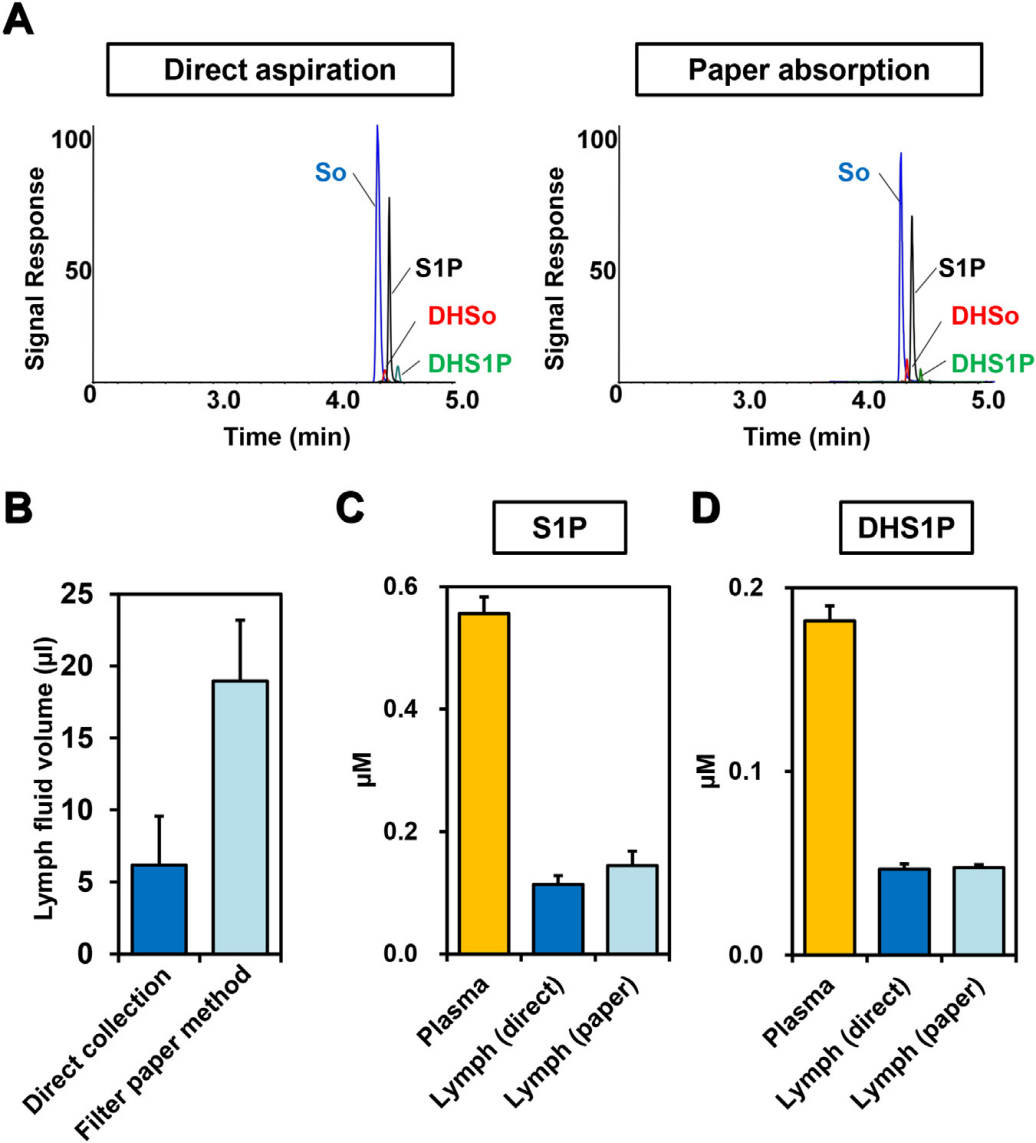
Levels of S1P and dihydro-S1P (DHS1P) in lymphatic fluid measured by mass spectrometry. (A) Detection of sphingoid bases. Extracted ion chromatograms for LC-ESI-MS/MS reverse-phased separation of sphingoid bases in the lymphatic fluid collected by direct aspiration (left) or paper absorption (right) are shown. (B) Volumes of lymphatic fluid collected by the direct method (blue bar) and the filter paper method (light blue bar) are shown. (C and D) Levels of S1P and DHS1P in plasma (yellow bars), or lymphatic fluid collected by the direct method (blue bars) or by the filter paper method (light blue bars) were measured by mass spectrometry. Data shown are mean ± SD (n = 3).

S1P levels were measured by mass spectrometry in the lymphatic fluid collected either by direct aspiration or by paper absorption (Fig. 2A). The volume of the lymphatic fluid collected by the paper absorption method was at least three times greater than that collected by direct aspiration (Fig. 2B). S1P and dihydro-S1P (DHS1P) levels in lymphatic fluid from both paper absorption and direct aspiration methods showed consistent results with minimal variation (Fig. 2C and D). In mice, S1P concentrations in lymphatic fluid were in the range of 0.1 to 0.3 μM (Fig. 2C), compared to more than 0.5 μM in plasma. The level of dihydro-S1P (DHS1P), which is generated by the de novo pathway and is also a ligand for all of the S1P receptors, was about 0.05 μM in lymphatic fluid and almost 0.2 μM in plasma (Fig. 1D). It is important to note that levels of S1P in lymphatic fluid collected by the filter paper method are in excellent agreement with previous reports on lymphatic fluid collected by the cannulation method [23, 28].

### 3.2 S1P gradient between blood, lymphatic fluid, and mesenteric lymph node (MLN)

**Fig. 3:**
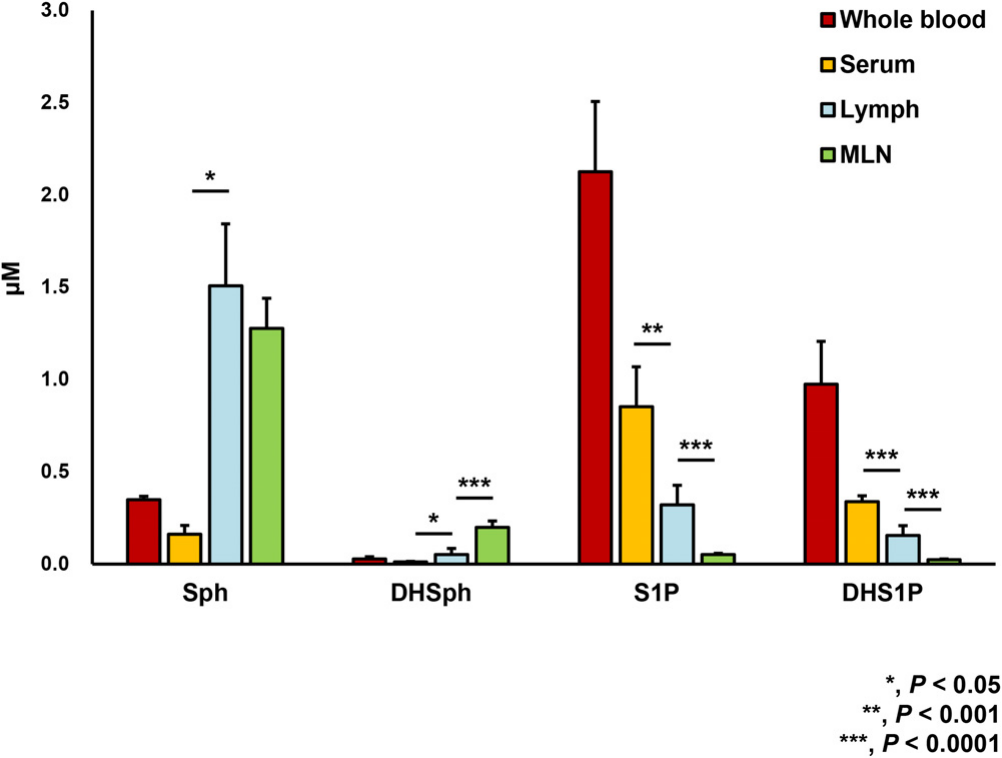
Levels of sphingolipids in whole blood, serum, lymphatic fluid (lymph), and mesenteric lymph node (MLN) from wild type (WT) mice. Levels of sphingosine (Sph), dihydro-Sph (DHSph), S1P, and dihydro-S1P (DHS1P) in whole blood, serum, lymph, and MLN from WT mice were determined by LC-ESI-MS/MS. Mean ± SD (n = 6). *, *P* < 0.05; **, *P* < 0.001; ***, *P* < 0.0001 for serum vs. lymph or lymph vs. MLN.

Next, we tested the theory of S1P gradient among serum, lymphatic fluid, and peripheral tissue such as MLN utilizing our new method to measure S1P in the lymphatic fluid. Levels of Sph, DHSph, S1P, and DHS1P in whole blood, serum, lymph, and MLN from WT mice were determined by LC-ESI-MS/MS (Fig. 3). S1P and DHS1P levels in the lymphatic fluid are significantly lower than those in serum. On the other hand, S1P and DHS1P levels in lymphatic fluid are significantly higher than those in MLN. Of note, both Sph and DHSph levels in lymph are significantly higher than those in serum (Fig. 3). DHSph, but not Sph, in the MLN showed significantly higher than those in lymphatic fluid (Fig. 3).

### 3.3 Effect of deletion of SphK2 on sphingolipid levels in blood, lymphatic fluid, and MLN

**Fig. 4:**
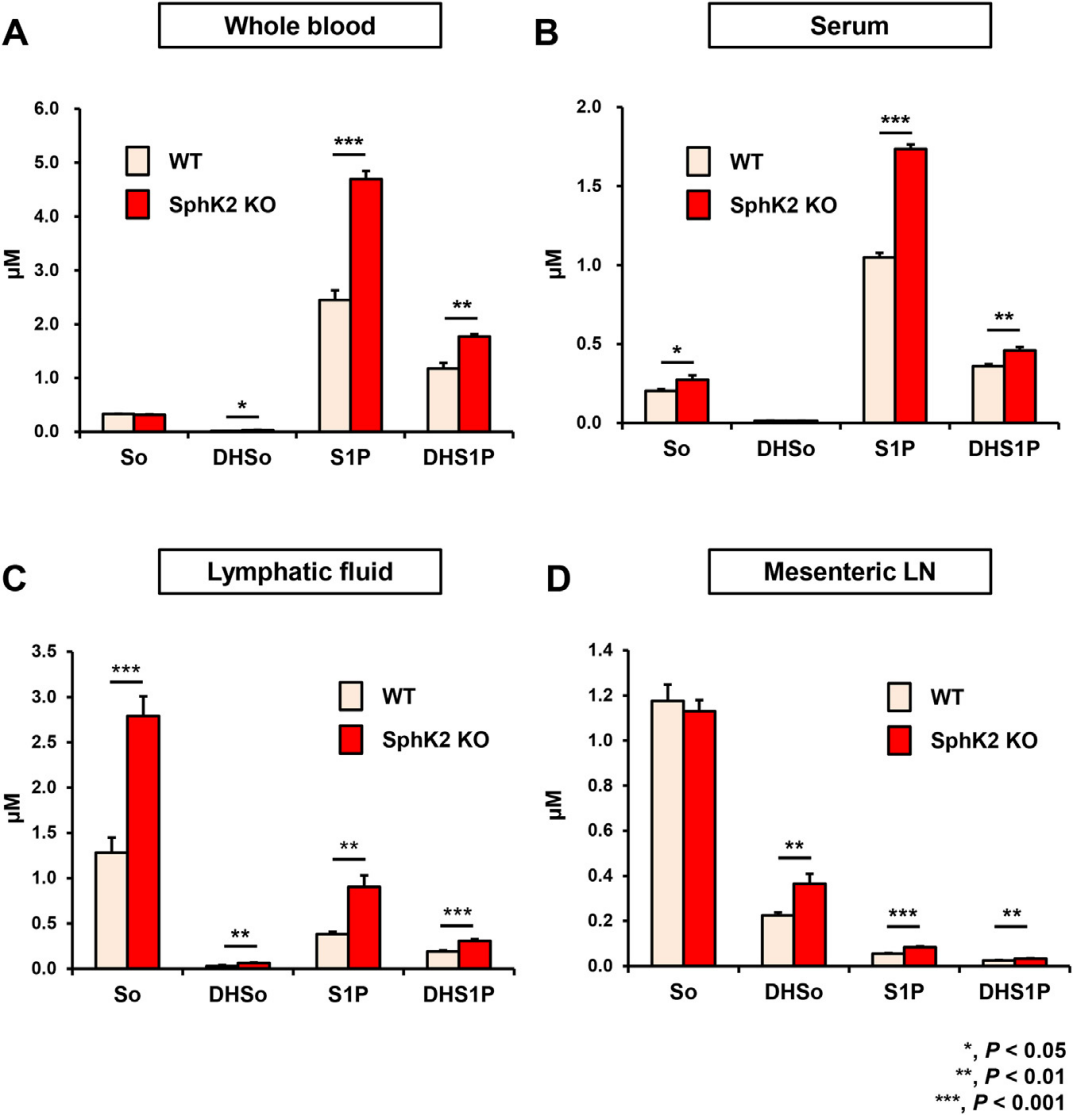
Levels of sphingolipids in lymphatic fluid from SphK2^−/−^ mice and littermate WT mice. Levels of sphingosine (Sph), dihydro-Sph (DHSph), S1P, and dihydro-S1P (DHS1P) in lymphatic fluid in (A) whole blood, (B) serum, (C) lymphatic fluid, (D) mesenteric lymph node from SphK2^−/−^ and their littermate WT were determined by LC-ESI-MS/MS. Mean ± SEM (n = 4). *, *P* < 0.05; **, *P* < 0.01; ***, *P* < 0.001.

The relative contributions from each of the SphK isoforms to lymphatic fluid and tissue levels of S1P have not been fully elucidated. To this end, we next examined the effects of deletion SphK2. In agreement with previous reports [12, 28], S1P and dihydro-S1P levels in whole blood and serum of SphK2 knockout mice were higher than their WT littermates, most likely due to compensatory up-regulation of SphK1 [12] (Fig. 4A and B). Levels of Sph and DHSph in whole blood and serum are much lower than the phosphorylated sphingoid bases in the SphK2 knockout and WT mice, as was previously reported [7, 28, 29, 30, 31]. Interestingly, levels of S1P and dihydro-S1P in the lymphatic fluid as well as mesenteric lymph node were significantly increased in SphK2 mice compared to their littermates (Fig. 3B). These results suggest the roles of SphK2 and/or SphK1 to regulate the levels of S1P and DHS1P in lymphatic fluid and the peripheral tissue.

## 4 Discussion

High levels of S1P in blood and lymphatic fluid are critical for maintenance of tone and integrity of the vascular endothelium. The S1P gradient between high levels in the circulation and the low levels in tissues due to the presence of S1P degrading activity from phosphatases and S1P lyase is important for immune cell trafficking [17, 32]. In this regard, it has been generally assumed that S1P levels in lymphoid tissues are very low so that S1PR1 on lymphocytes can sense the S1P gradient as they exit into the blood or lymphatic fluid. However, only a handful of studies have reported S1P levels in lymphatic fluid [7, 23, 28]. To overcome the hurdles for S1P measurement in the lymphatic fluid, we have established simple and reliable methods to collect adequate amounts of lymphatic fluid to enable the measurement of levels of bioactive sphingolipids by LC-ESI-MS/MS.

The most commonly described technique for lymphatic fluid collection is by cannulation of lymphatic vessels [23, 28, 33, 34]. The cannulation method has several advantages: long-term continuous observation is possible, a higher amount of fluid can be obtained, and importantly, contamination from other body fluids is relatively low. However, this method requires special equipment and is technically challenging in mice and therefore the majority of studies have used larger animals [33, 34]. On the other hand, direct aspiration with a needle is less difficult to perform, but usually collapses the vessel resulting in a lower yield. The challenge of obtaining enough fluid is met by our filter paper absorption method, which does not require special equipment, is relatively easy to perform when adequate exposure of the vessel is obtained, eliminates the effect of irritation from the cannula on the lymphatic vessel wall, and allows for immediate sampling. Since the paper absorption technique is easy to perform, any person who works on animal study can use it. Indeed, not only experienced surgeons, but also surgical residents and technicians were able to perform this technique to collect lymph. It is vital to avoid contamination with blood, which contains high concentrations of S1P. Nevertheless, if it occurs, it can readily be detected by the appearance of the red color of hemoglobin on the filter paper. Contamination with other body fluids could be another concern. However, this is unlikely since the level of S1P in lymphatic fluid collected by the direct filter absorption method is very similar to that obtained by needle aspiration, which has very low possibility of contamination. As a further validation of our method, previous reported S1P levels in lymphatic fluid collected by cannulation [23, 28] are in excellent agreement with the data from the filter paper absorption method. Therefore, we suggest that this paper absorption method is a reliable and simple method for collection of lymphatic fluid from mice. Although this collection method via the cisterna chili is likely only applicable for animal models, it is expected to be a valuable tool for studies to elucidate the functions of S1P in inflammatory diseases, lymphatic vascular disorders, and cancer invasion into the lymphatic system.

SphK1 which is localized mainly in the cytosol and translocated to the plasma membrane upon stimulation by cytokines and growth factors, is important for export of S1P and regulation of its extracellular levels [8, 35]. In contrast, SphK2 is localized to several intracellular organelles including the endoplasmic reticulum, the mitochondria, and the nucleus where it produces S1P that regulates intracellular functions. [36, 37, 38, 39]. Interestingly, knocking out SphK2 increased levels of S1P and dihydro-S1P in both blood and lymphatic fluid. It has been reported that mice lacking both SphK1 and SphK2 in endothelial cells have a loss of S1P in lymphatic fluid while maintaining normal plasma S1P, suggesting that SphKs in lymphatic endothelial cells are the source of S1P in lymphatic fluid [24]. In contrast to lymphatic fluid, serum S1P is derived predominantly from hematopoietic cells, with red blood cells playing a major role [23, 40]. Higher levels of S1P in both serum and lymphatic fluid in SphK2 knockout mice could be explained by upregulation of SphK1 in both hematopoietic cells and endothelial cells. On the other hand, considering that the sources of S1P in serum and lymphatic fluid are different, it is possible that global knockout of SphK2 affects production of S1P in the hematopoietic cells, but induces some compensatory mechanism either in lymphatic endothelial cells or in upstream tissue to maintain S1P level in lymphatic fluid. Further studies are needed with tissue specific knockout of SphK1 and/or SphK2 to explore the mechanisms by which levels of S1P in lymphatic fluid are regulated.

## 5 Conclusions

We determined the levels of S1P in lymphatic fluid, which is lower than blood and higher than lymph nodes. In agreement with the previous theory, our results confirm the “S1P gradient” among blood, lymphatic fluid and the peripheral lymphatic tissues. Convenient methods for collection and measurement of sphingolipids in lymphatic fluid are expected to provide new insights on functions of sphingolipids.

## Declarations

### Author contribution statement

Masayuki Nagahashi: Conceived and designed the experiments; Performed the experiments; Analyzed and interpreted the data; Wrote the paper.

Akimitsu Yamada, Tomoyoshi Aoyagi: Performed the experiments.

Jeremy Allegood: Performed the experiments; Contributed reagents, materials, analysis tools or data.

Toshifumi Wakai: Analyzed and interpreted the data.

Sarah Spiegel: Analyzed and interpreted the data; Wrote the paper.

Kazuaki Takabe: Conceived and designed the experiments; Analyzed and interpreted the data; Contributed reagents, materials, analysis tools or data; Wrote the paper.

### Funding statement

This work was supported by the Japan Society for the Promotion of Science (JSPS) Grant-in-Aid for Scientific Research Grant Number 15H05676 and 15K15471 for Masayuki Nafahashi, and 15H04927 and 16K15610 for Toshifumi Wakai. Masayuki Nagahashi is also supported by the Uehara Memorial Foundation, Nakayama Cancer Research Institute, Takeda Science Foundation, and Tsukada Medical Foundation. Kazuaki Takabe is supported by NIH/NCI grant R01CA160688 and Susan G. Komen Investigator Initiated Research Grant IIR12222224.

### Competing interest statement

The authors declare no conflict of interest.

### Additional information

No additional information is available for this paper.
